# Analysis of the Effect of Surface Preparation of Aluminum Alloy Sheets on the Load-Bearing Capacity and Failure Energy of an Epoxy-Bonded Adhesive Joint

**DOI:** 10.3390/ma17091948

**Published:** 2024-04-23

**Authors:** Barbara Ciecińska, Jacek Mucha, Łukasz Bąk

**Affiliations:** 1 Department of Manufacturing Processes and Production Engineering, Rzeszow University of Technology, 35-959 Rzeszow, Poland; bcktmiop@prz.edu.pl; 2 Department of Mechanical Engineering, Rzeszow University of Technology, 35-959 Rzeszow, Poland; 3 Department of Materials Forming and Processing, Rzeszow University of Technology, 35-959 Rzeszow, Poland; lbak@prz.edu.pl

**Keywords:** laser texturing, interfacial bonding strength, Industry 5.0, surface properties, lap-shear strength

## Abstract

Surface preparation is an important step in adhesive technology. A variety of abrasive, chemical, or concentrated energy source treatments are used. The effects of these treatments vary due to the variety of factors affecting the final strength of bonded joints. This paper presents the results of an experimental study conducted to determine the feasibility of using fiber laser surface treatments in place of technologically and environmentally cumbersome methods. The effect of surface modification was studied on three materials: aluminum EN AW-1050A and aluminum alloys EN AW-2024 and EN AW-5083. For comparison purposes, joints were made with sandblasted and laser-textured surfaces and those rolled as reference samples for the selected overlap variant, glued with epoxy adhesive. The joints were made with an overlap of 8, 10, 12.5, 14, and 16 mm, and these tests made it possible to demonstrate laser processing as a useful technique to reduce the size of the overlap and achieve even higher load-bearing capacity of the joint compared to sandblasting. A comparative analysis was also carried out for the failure force of the adhesive bond and the failure energy. The results show the efficiency and desirability of using lasers in bonding, allowing us to reduce harmful technologies and reduce the weight of the bonded structure.

## 1. Introduction

Adhesive bonding is a technique for joining a variety of structural materials, which, despite a number of disadvantages, is still valid and widely used. Typical disadvantages that can be mentioned are the low strength of the joints at higher temperatures (most often, the maximum temperature is 200 °C), the low strength of the adhesive, the need to develop the surface to improve the conditions of mechanical adhesion, and the cumbersome nature of the typical methods of imparting greater roughness, i.e., sandblasting, degreasing, and chemical etching [1,2,3]. Difficulties in achieving a satisfactory bond can also include the setting time of the adhesive, which, if longer, also affects the lengthening of the process, and the open time, which, in turn, varies depending on the type of adhesive; it can be very short, ranging from a few minutes, as in the case of cyanoacrylate and some epoxy adhesives, and several minutes, as in the case of methacrylate and polyurethane adhesives, to more than an hour in the case of many epoxy adhesives. This feature has a key impact on the bonding process, especially on the dosage of the mixture and the possible loss of properties [4,5,6].

However, despite the aforementioned inconveniences, bonding is a willingly used method of joining structural components. This is because it makes joining diverse materials, such as steel, aluminum and its alloys, titanium and its alloys, polymers, and composites, in homogeneous and hybrid joints possible [7,8,9,10]. An additional advantage is the possibility of joining elements against which welding cannot be applied (e.g., some corrosion-resistant steels) or for which it would be unfavorable to make holes as it would weaken the structure (as in riveting). Bonding, moreover, does not create an unfavorable state of stress, as with welding or sealing. In addition, bonding prevents the formation of bimetallic corrosion, which occurs in joining metals of different potentials (e.g., steel with copper or its alloys) [11,12,13]. The advantages of this technology include, in particular, strength, versatility, the ability to join thick parts to thin parts, improved joint stiffness, tightness, light weight (due to the lack of additional load in the form of weld material or rivets), and the ability to control technological parameters (type of adhesive, curing temperature, thickness of the adhesive layer, preparation method, and geometric structure of the bonded surfaces) in order to achieve the maximum joint strength [14,15,16]. From a manufacturing point of view, the bonding process is relatively inexpensive, components with high manufacturing inaccuracy can be bonded, the joint does not require finishing operations, it is aesthetically pleasing, and above all, it is practical for automation and robotization, which is particularly useful in the context of modern developments referred to as Industry 5.0 [17].

Due to the variety of bonded materials and adhesives, a common issue is the generation of specific adhesion conditions. This issue can be analyzed in terms of mechanical adhesion, and then mechanical and chemical treatments can be applied (sandblasting, grinding, milling, and electroplating baths), as well as specific adhesion. Then, the treatment is performed to activate the surface, that is, to create a state of high surface energy and very good wettability [18,19]. This is achieved by applying a primer layer, ozonation, and plasma treatment. A third way is to modify the chemical composition and ratio of the adhesive itself [4].

Metal parts are usually characterized by a smooth and coherent surface structure. This surface is often covered with a layer of dirt and grease—residues from previous processing, such as through rolling. Sometimes, it is specially coated with a layer of anti-corrosion preparation. The process of preparation for bonding requires the removal of these layers with special preparations (gasoline, kerosene, and trichloroethylene), washing with water, and drying. After degreasing, washing, and drying, the surface is abraded manually with sandpaper or corundum in ejector chambers; then, it is washed again (e.g., in acetone) and dried [20,21]. Only then is it suitable for bonding. Acid or alkaline baths of varying composition containing toxic, hazardous components (e.g., H_2_SO_4_, CrO_3_, Na_2_CrO_7_, K_2_CrO_7_, and NaOH) are also used [22,23]. The presence of such substances in the technological process is currently very problematic for environmental reasons; although the effects of such processing methods are very beneficial because the produced surface layer is porous and very permanently bonded to the substrate, which significantly improves the adhesion conditions and strength of the joints [23], due to the environmental burden of waste in the form of contaminated and used electroplating baths, as well as the significant consumption of clean water for the washing and drying of components, it is evaluated negatively; therefore, a change in technology towards waste-free and environmentally benign technologies is desired [24,25].

Surface activation via ozonation/UV or plasma treatment is used most often for polymer surface preparation and can be assisted by primer application [26,27]—or, alternatively, can be combined with cumbersome sandblasting [28]—and the results may not be satisfactory anyway. In addition, such processes require specialized equipment. Similarly, the adhesive mixture can be modified with varying results, e.g., by dosing the hardener accordingly and increasing the brittleness of the joints [29] and modifying the composition with nanofillers [30,31]. There is also a new concept of bonding that mimics nature—the use of coating with a special layer of polyamide covered with a thin layer of photosensitive emulsion with synthetic tabs. This idea is based on the observed foot of a gecko, which is covered with dry, flexible hairs (setae), the ends of which are divided into nanoscale structures (spatulae), similar to actual spatulas. The number of these spatulae and their nearness means that only van der Waals forces are sufficient to provide the required adhesion force [32,33]. 

This is the research direction of so-called biomimetic adhesives, developed today with further inspiration from nature: a structure along the lines of a gecko’s feet is being developed into a multi-layered, self-peeling, switchable dry/wet adhesive, combining thermoactive hydrogel layers inspired by both mushroom-structured arrays and mussel-inspired copolymer adhesive coatings [34]. As attractive and scientifically promising as they may be, they are currently yet to be implemented on a wider scale. 

This article presents experimental research on joints made of aluminum and its alloys. These materials are common in automotive and aerospace applications, among others, but require greater surface roughness before bonding [35,36,37]. Sandblasting, which is typical for these materials, has been replaced by laser processing due to a number of advantages of the laser beam, such as the consistency of the energy beam; the ease of controlling parameters such as wavelength, beam power, and pulse frequency; and the speed of the beam relative to the surface. In addition, the impact of the concentrated energy beam as a tool is stable over time and repeatable, and material heating can be reduced to the necessary minimum [38,39]. In manufacturing, laser processing can be easily automated and robotized, and laser equipment can be integrated into computerized and flexible production lines due to the fact that the controller software used can be compatible with the other equipment in the line [40]. The integration of laser equipment is therefore possible in the context of Industry 5.0. Currently, many research centers are working on the laser texturing of surfaces before bonding [41,42,43].

The experimental research presented in this article was undertaken to investigate whether it is possible to further develop the technology of preparing the surface of chosen materials before bonding. The purpose of this study was to verify the effectiveness of laser surface texturing and to analyze this technology in the context of the gluing result expressed in terms of the load-bearing capacity of a single-lap joint. The results of bonding laser-prepared surfaces according to the selected processing scheme were compared with the results of bonding rolled and sandblasted surfaces. In addition, in search of an answer to the question of whether the laser processing technology will require an increase in the area of the joint, or if, on the contrary, it will be possible to glue the structure with a smaller overlap the load-bearing capacity of the joints was examined in the context of the size of the overlap.

## 2. Materials and Methods

### 2.1. Sheet Metals and the Adhesive

Sheets of aluminum and its alloys were used for experimental analysis of the effect of surface preparation and overlap size on the load-bearing capacity of the adhesive joint. Three grades of material were used: aluminum EN AW-1050A and the alloys EN AW-2024, EN AW-5083 (by EN [44]; respectively: Al99.5, AlCu4Mg1, and AlMg4.5Mn0.7). The basic chemical composition is summarized in Table 1, while the mechanical properties are listed in Table 2.

Araldite 2014-2 [46], an adhesive with the properties declared by the manufacturer according to ISO 527-2 [47], was used to make the adhesive bond. The adhesive is a thixotropic, two-component, epoxy-based binder. It is designed for bonding various types of materials, including metals, polymers, and composites. The manufacturer’s declared bonding time for the adhesive is 180 min, while the typical time to achieve minimum shear strength above 10 MPa for room temperature (23 °C) was 480 min (8 h). The basic characteristics of the adhesive are shown in Table 3.

### 2.2. Surface Preparation for Adhesive Bonding

The surfaces of the overlap to be used to apply the adhesive were prepared in different ways. As reference samples for determining the volume and nature of the change in the load-bearing capacity of the joints, the surfaces were left smooth after rolling (surface condition variant T.0). 

Other variants created by texturing the surface before adhesive bonding were made via the following processes:

- Sandblasting (T.I);
- Laser beam surface treatment with fixed laser parameters and beam pass spacing (T.II).

Samples with dimensions of 25 × 100 mm were cut from 1 mm-thick sheets. The samples for the T.0 variant were degreased with aliphatic hydrocarbon-based agent C_n_H_2n_ (C_6_H_14_) then washed with deionized water and dried. The surfaces of the sandblasted samples (variant T.I) were cleaned with demineralized water and dried in air at 21 ± 1.5 °C. In contrast, surfaces made via laser treatment (T.II) were dusted off with compressed air.

Sample preparation in the T.I variant—that is, by sandblasting—was performed in an ejector chamber using fine-grained F280-type electrocorundum with an average size of 45 ± 4 µm. The chemical composition of the micropowder composition is summarized in Table 4.

**Figure 1:**
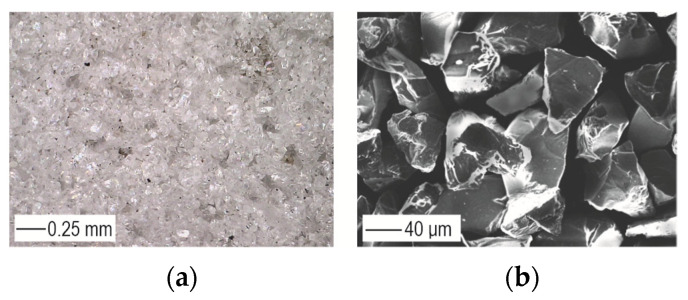
Macro photograph of abrasive powder (**a**) and grain morphology of F280 electrocorundum (**b**).

**Figure 2:**
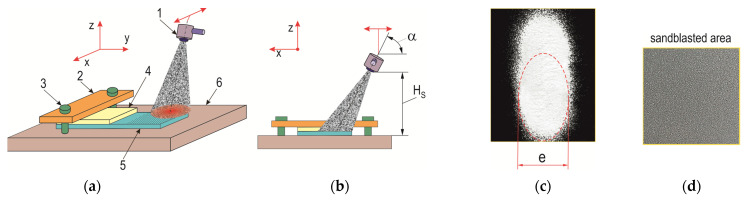
Texturing with an electrocorundum jet of the overlap surface before bonding: (**a**) conceptual scheme; (**b**) inclination of the abrasive jet with regard to the sheet (1—end of the nozzle; 2—pin; 3—connectors; 4—shielding sheet; 5—sample of the sheet to be bonded; 6—plate); (**c**) determination of the trace of the effect of the electrocorundum; (**d**) example of the surface of an aluminum alloy sheet after texturing.

The hardness of the electrocorundum was 9.0 on the Mohs scale, and the particles were characterized by an irregular shape and sharp edges (Figure 1). The samples were mounted on a flat surface for processing; the part to be bonded was exposed and subjected to an abrasive jet at a pressure of *p_s_* = 0.3 MPa (Figure 2a). The angle of inclination of the abrasive jet *α* = 45° and the distance of the nozzle outlet from the sandblasted surfaces (*H_s_* = 100 mm) were determined experimentally (Figure 2b); the abrasive jet at such parameters had a width of *e* = 22 ± 1.5 mm (Figure 2c). An example photograph of the surface after sandblasting is shown in Figure 2d.

**Figure 3:**
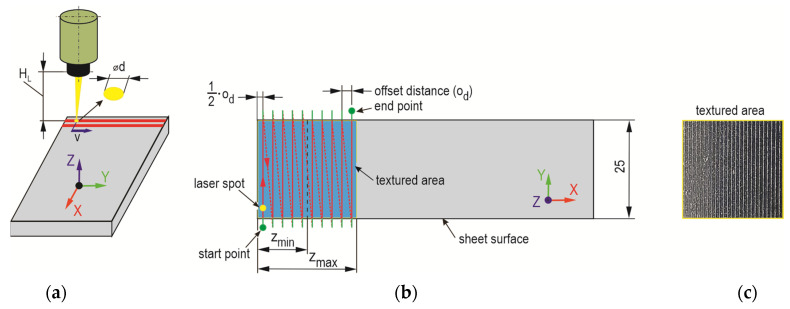
The method of laser processing: (**a**) the main parameters of the laser beam; (**b**) the parameters of the path of the laser beam passes (*z*—the length of the overlap (*z_min_* = 8 mm; *z_max_* = 16 mm); *ϕd*—laser beam spot diameter; *H_L_*—focal length for a spot *ϕd* (*H_L_* = 320 mm)); (**c**) example of aluminum alloy sheet surface after laser texturing. The red solid line indicates the trace of the laser beam interaction, the red dash line indicates the beam return path to the beginning of the next scan line (without processing), the green solid line is the course of the laser beam outside the over-lap area.

Laser beam texturing of T.II variant samples was performed according to the established scheme (Figure 3). In order to increase the peel resistance of the adhesive layer from the smooth surface of the sheets (not chemically etched), a transverse line arrangement of laser beam modification of the overlap surface was adopted. This choice was based on the experience gained from previous experimental work described in [48], where such a texturing arrangement was favorable. The processing was carried out with a pulsed single-mode fiber laser on ytterbium with a nominal power of 20 W, a wavelength of 1060–1080 nm, a spot size of 2–3 μm, a maximum energy in the pulse of 0.8 mJ, and a pulse duration of 200 ns (Laser G4, source and fiber—SPI, TRUMPF Group, Southampton, UK; equipment—DK Lasertechnik, Cracov, Poland). On the surface of the metal sheets, laser beam passes were made 10 times at a speed of 200 mm/s with a pulse frequency of 45 kHz and maximum nominal power of 20 W. The spacing between the paths of the laser beam passes was *o_d_* = 0.5 mm. The processing was performed in air without a protective atmosphere.

### 2.3. Evaluation of the Geometric Structure of the Surface

In order to determine the basic parameters of the evaluated areas prepared for bonding, surface microtopography measurements were carried out. Identification of the average value of surface roughness parameters *Ra* and *Rz* for the three cases of surface preparation for bonding was performed using a 3D Taylor Hobson Talyscan 150 Laser Scanner (Taylor Hobson, Leicester, UK). To visualize the difference in SGP between the micropowder-textured overlap surface and the laser-beam-modified surface texture, representative areas were scanned according to ISO 4288 [49]. The visualization of the unevenness is presented in a smaller area of 2 mm × 2 mm. The most important results representing stereometric parameters determined based on ISO 25178-2 [50] were selected. On the basis of preliminary analyses, the value of the limiting wavelength of the low-pass filter *λ_s_* was set at 0.8. Image observations using a VHX7000 optical microscope (KEYENCE INTERNATIONAL, Mechelen, Belgium) were used to characterize the transverse shape of the joint.

### 2.4. Sample Joint Preparation

**Figure 4:**
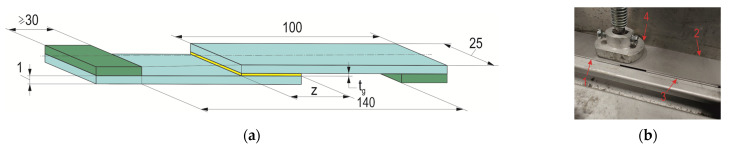
Single-lap joint: (**a**) appearance and characteristic dimensions (in mm); (**b**) the method of pressing the sample. 1—bottom overlap sheet, 2—upper overlap sheet, 3—stabilizing plate, 4—pressure plate.

**Figure 5:**
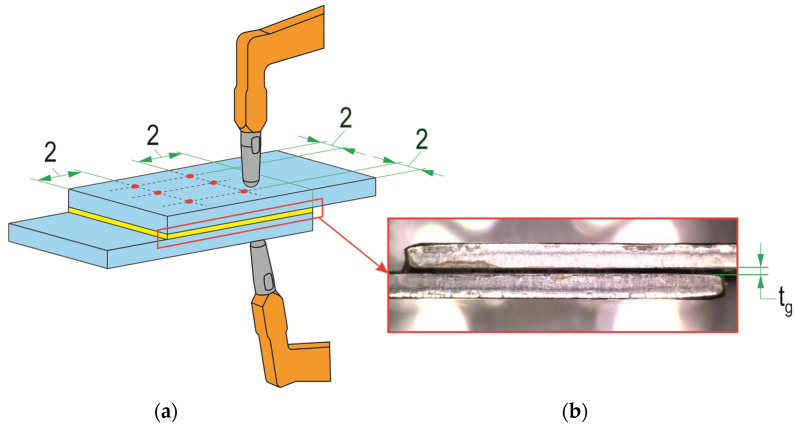
Checking the thickness of the adhesive layer in the joint: (**a**) indirect method of measuring the average thickness of the adhesive; (**b**) removal of the joint with Araldite 2014-2 adhesive (*t_g_*—thickness of the adhesive layer).

For the tests, single-lap rolled sheet joints were made from the previously mentioned materials with the dimensions of the glued sample shown in Figure 4a. Reference samples (T.0) had an overlap in accordance with the standard [42], i.e., 12.5 mm. The T.I and T.II samples were made with overlaps of different sizes, as shown in Table 5. The gluing was performed in an air-conditioned room at a temperature of 22 ± 0.5 °C and a humidity of 40 ± 2%. The lap joints were pressed with a weight of 1 kg for 24 h (Figure 4b). Strength tests of the joints were performed after another 24 h of the adhesive drying. In order to minimize the range of scatter in the results, excess glue at the lap zone was removed with a 3 mm-diameter cutter. The thickness of the adhesive layer was checked with Mitutoyo 209–572 measuring tentacles (Mitutoyo, Wroclaw, Poland) with a pitch of 1 μm. The thickness of the adhesive layer was measured at 6 evenly spaced points (Figure 5a). An example of the appearance of the adhesive layer between the sheets is shown in Figure 5b. Each joint variant was performed for 5 repetitions, and the average thickness of the adhesive was *t_g_* = 0.15 mm.

### 2.5. Quasi-Static Lap-Shear Tensile Test

**Figure 6:**
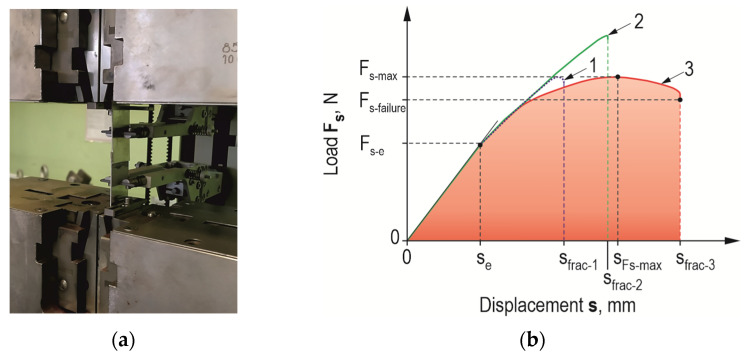
Adhesive lap joint (**a**) during strength test; (**b**) joint load curve and characteristic parameters.

Load tests of the joints were performed on a ZWICK/ROELL Z100 testing machine (Zwick Roell, Wroclaw, Poland) equipped with an extensometer system (Figure 6a). The force-recording head was characterized by a maximum measurement force of up to 100 kN and a maximum measurement error of 0.5% of the measurement range (class 1). An extensometer with a measuring range of 50 mm and displacement registration accuracy of class 0.5 was used to measure displacement. Adhesive joints were subjected to shear by extension of the lap joint (Figure 6). The displacement speed of the crosshead, forcing the deformation of the joint, was 5 mm/min. For the obtained force waveforms as a function of displacement, the results of the maximum load capacity were developed and the work of failure of the adhesive joint *E_t_* was calculated (Figure 6b), according to the following relation:

(1) $$E_{t}=\int_{s=0}^{s_{frac}}F_{s}\cdot ds.$$

The static shear strength *R_m_* of the joint was calculated according to the relation, in which *F_s−max_*—the maximum force that was recorded in the experiment and transmitted through the joint, in N; *A*_0_—nominal overlap area, in mm:

(2) $$R_{m}=\frac{F_{s-max}}{A_{0}}.$$

In the case of weak adhesion of the adhesive to the substrate, the maximum displacement for which the joint fails completely is much smaller (curve 1—in the Figure 6b) than that for an overlap with a machined surface (curve 2—in the Figure 6b) for the same material. Curve 3 (in the Figure 6b) represents the case of the accumulation of the adhesive and cohesive failure mechanism (after the maximum load force on the joint is reached). The latter can occur when the same adhesive is used, and the appropriate treatment is applied to give the relief above the surface of the sheet.

## 3. Results and Discussion

### 3.1. Surface Texture and Morphology of the Traces after Laser Treatment

**Figure 7:**
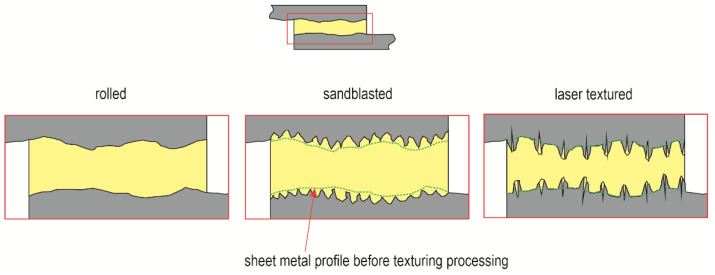
Diagram of the surface structure of the overlap and the means of its filling with adhesive.

The surface of the overlap after sandblasting was characterized by microcavities spread over the entire surface, while after laser texturing, the local microcavities were arranged in relation to each other in a specific manner resulting from the method of processing (Figure 7). For the initial surface after rolling, the values of surface roughness parameters *Ra* and *Rz* were also checked, and statistical parameters were determined (Table 6).

**Figure 8:**
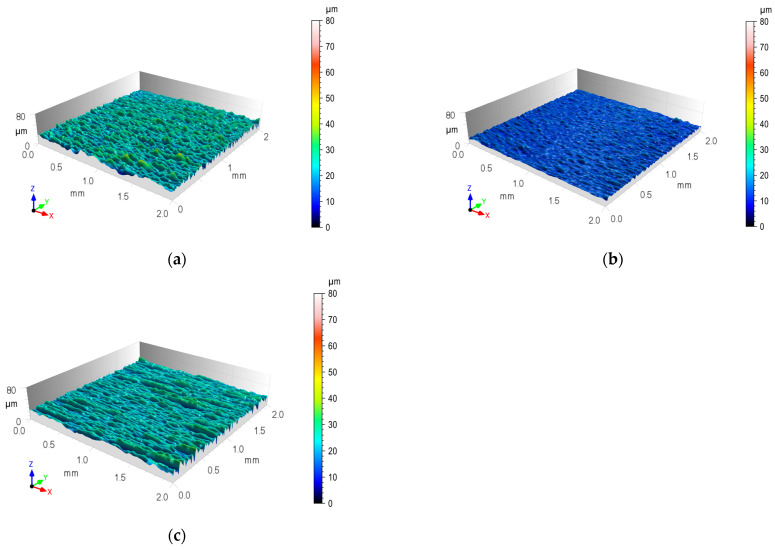
Isometric view of the contour map of the output surface structure after rolling (T.0): (**a**) EN AW-1050A; (**b**) EN AW-2024; (**c**) EN AW-5083.

**Figure 9:**
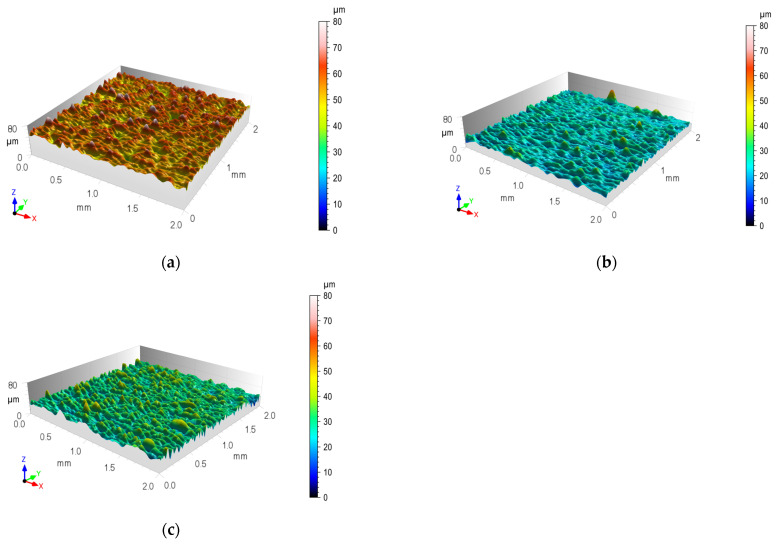
Isometric view of contour map of micropowder-textured lap surface structure (T.I): (**a**) EN AW-1050A; (**b**) EN AW-2024; (**c**) EN AW-5083.

**Figure 10:**
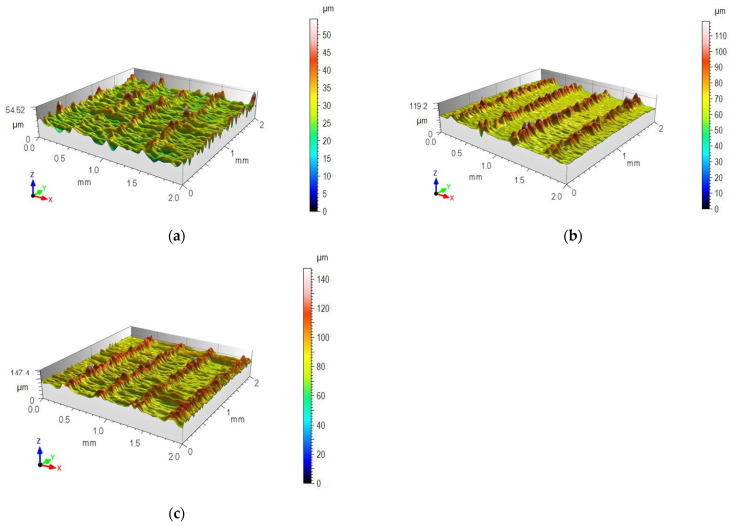
Isometric view of contour map of laser-textured lap surface structure (T.II): (**a**) EN AW-1050A; (**b**) EN AW-2024; (**c**) EN AW-5083.

**Figure 11:**
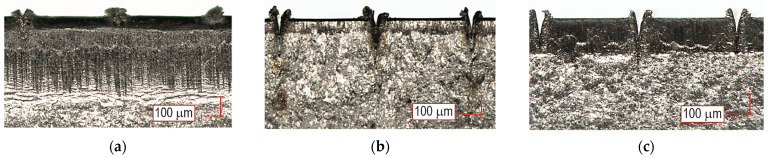
Morphology of cavities made by laser beam for alloy: (**a**) EN AW-1050A; (**b**) EN AW-2024; (**c**) EN AW-5083.

The geometric structures for the T.0, T.I, and T.II variants of the overlap surface are shown in Figure 8, Figure 9 and Figure 10. The initial overlap surface was characterized by a directional arrangement of irregularities (Figure 8). The direction of their arrangement is due to their processing by rolling. The application of sandblasting reduced the directionality of the structure, and the height of the irregularities increased (Figure 9). In this case, the top layer of the existing aluminum oxides was removed, and no chemical surface treatment was applied. Laser treatment created transverse indentations in relation to the longitudinal texture after rolling. Microgrooves with a very narrow gap were created on the surface. The laser profilometer works by reconstructing surface information from reflected light. The structure of the ejected liquid material on the edges of the grooves was well reproduced. Examples of the transverse outlines of the indentations for the aluminum alloys used are shown in Figure 11. The most regular and symmetrical indentation was obtained for the EN AW-5083 alloy.

### 3.2. Load-Bearing Capacity of Lap Joints

**Figure 12:**
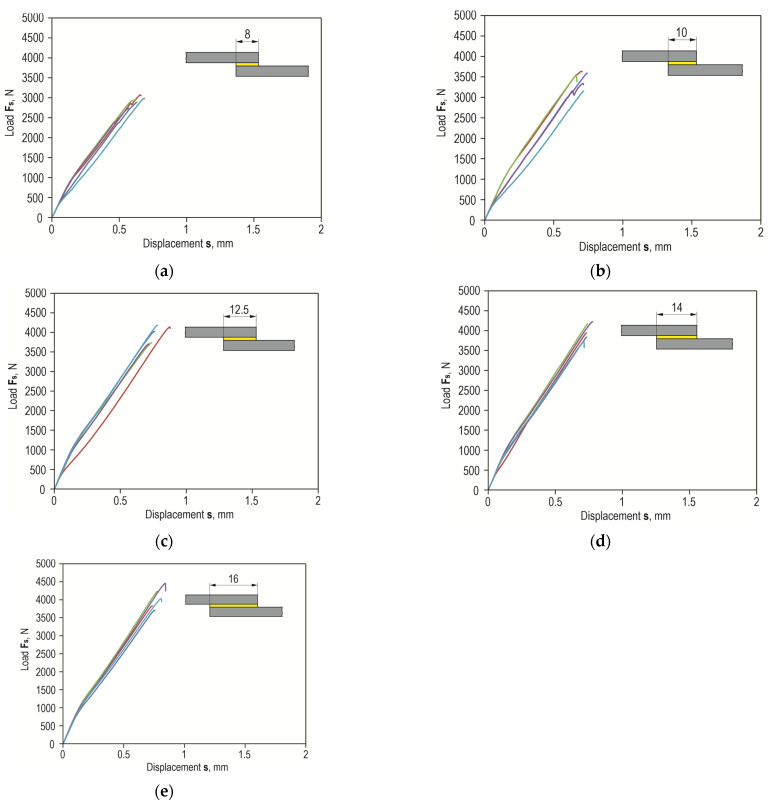
Examples of overlap shear curves of Araldite 2014-2 adhesive joint with different overlap sizes z = 8, 10, 12.5, 14, 16 mm for EN AW-2024 sheet material (laser-treated lap surface—T.II)—(**a**–**e**).

**Figure 13:**
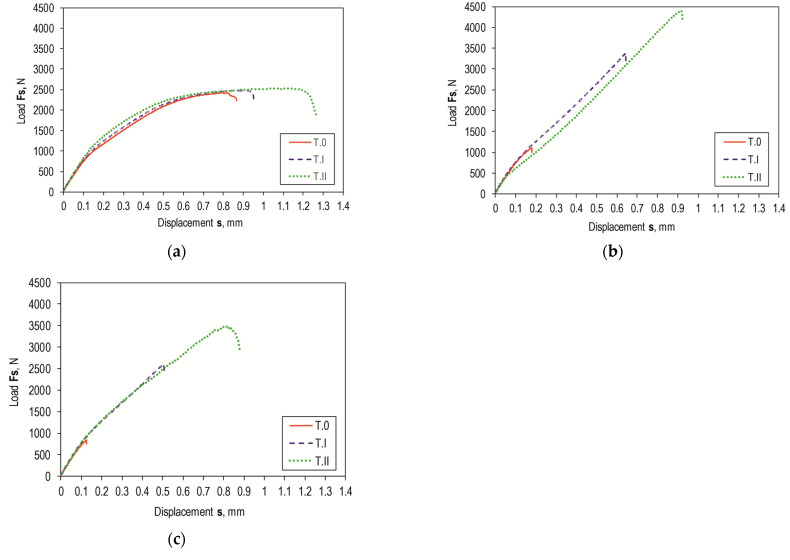
The impact of surface condition and texturing technology on the load-bearing capacity of a bonded joint for an aluminum alloy (*z* = 12.5 mm): (**a**) EN AW-1050A; (**b**) EN AW-2024; (**c**) EN AW-5083.

**Figure 14:**
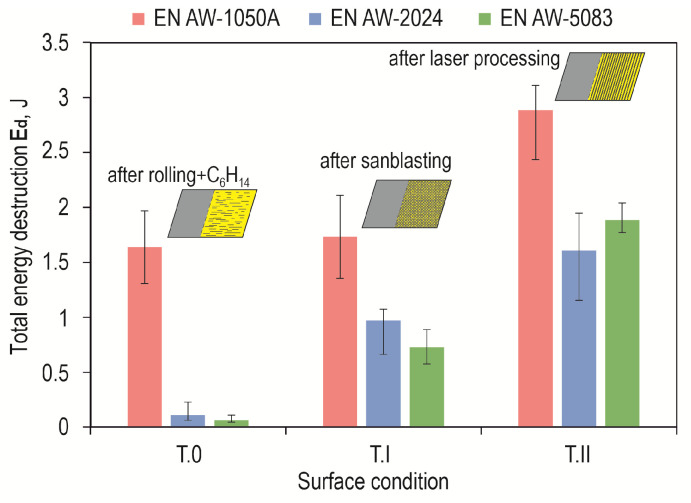
The average value of the failure energy of the lap joint (*z* = 12.5 mm) for the three tested materials and the varying condition of the lap surface.

Shear force waveforms as a function of displacement were recorded for all combinations of the lap joint with Araldite 2014-2 adhesive. Example shear curves for a series of joints with different overlaps for the sheet material (substrate for the adhesive) EN AW-2024 are in Figure 12, with a laser-treated lap surface. On their basis, selected parameters of joint strength were determined. Example load–displacement curves for the tested materials with a 12.5 mm overlap of the adhesive joint are summarized in Figure 13. Despite laser treatment of the surface of the EN AW-1050A sheet overlap, no significant increase in the joint’s maximum failure force was observed. The linear indentations produced increased the energy required to destroy the joint (Figure 13a). Damage accumulation occurred at a much higher displacement (1.2 mm). In the case of the EN AW-2024 alloy, the surface condition affected not only the maximum load-carrying capacity but also the failure energy (Figure 13b). The maximum load-carrying capacity of the joint after laser treatment compared to the untreated surface increased by about 285%. In contrast, the increase was just over 296% for EN AW-5083 material. The load-bearing capacity of the EN AW 2024 and EN AW 5083 material joints for the sandblasted surface was also significantly higher than for the untreated surface (Figure 13b,c). The connection with the laser-treated lap compared to the sandblasted one had an average maximum bearing capacity that was only 5% higher for the EN AW-1050A alloy. For the EN AW-2024 alloy, the increase in load capacity was already 28%, while for the EN AW-5083 sheet material, the load capacity was 35% higher. In each of the cases of joining materials, an increase in the failure energy of the joint was obtained after applying additional treatment to the overlap surface (Figure 14).

**Figure 15:**
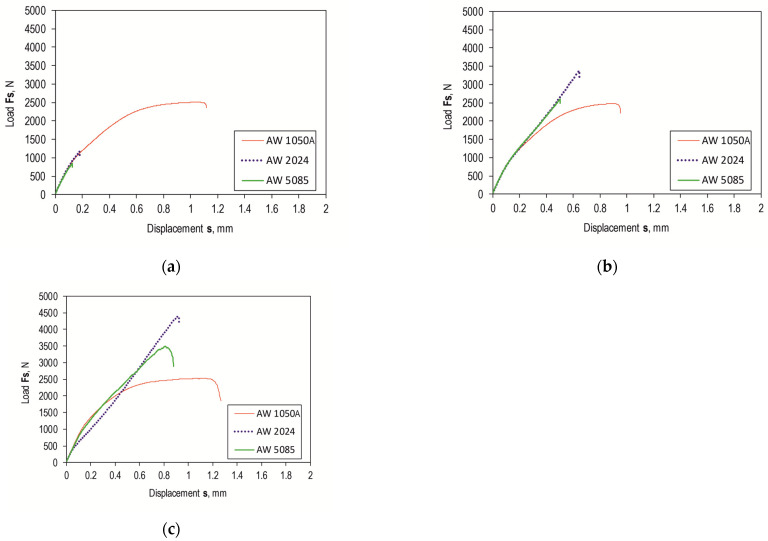
Effect of surface condition and texturing technology on bonded joint load capacity for 12.5 mm overlap: (**a**) T.0; (**b**) T.I; (**c**) T.II.

Lap joints for all three bonding materials, prepared via degreasing with C_6_H_14_ compound, showed the lowest load capacity (Figure 15a). As can be seen in Figure 15a, the highest load-bearing capacity of the joint was obtained for the adhesive substrate material with EN AW-1050A. The use of abrasive treatment of the overlap surface changed the load-bearing capacity of the joints (Figure 15b). The highest load carrying capacity of the joint in this case was already obtained for the joint with EN AW-2024. The use of laser treatment to create transverse grooves in relation to the effect of the loading force on the lap joint resulted in an increase in load-carrying capacity for EN AW-2024 and EN AW-5083. In contrast, for the EN AW-1050A alloy, the total failure energy of the joint increased without a clear increase in the load-carrying capacity of the joint (Figure 14 and Figure 15c).

**Figure 16:**
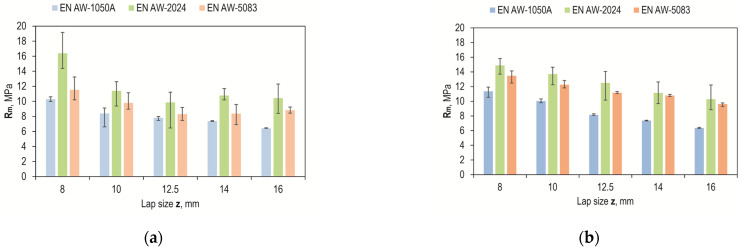
Effect of overlap size on maximum joint strength: (**a**) sandblasting; (**b**) laser texturing.

Figure 12 and Figure 15 show a comparison of the curves for the average value of the joint resistance. For joints with different overlap sizes (from 8 to 16 mm) treated by sandblasting and via laser beam, bar graphs were drawn for the average value of joint failure energy along with the maximum and minimum values (Figure 16).

The results of joint load capacity tests for different joint overlap sizes with sandblasted and laser-prepared surfaces, shown in Figure 16, indicate that the highest design joint strength was obtained for the EN AW-2024 alloy and an overlap of 8 mm. This was 16.4 MPa for the sandblasted surface and 14.9 MPa for the laser-textured surface. Joints with sandblasted surfaces had the lowest strength for a 12.5 mm overlap (Figure 16a), but the spread of values from the average value was significant. In the case of laser processing, the scatter of values from the average value was smaller, which was observed for all materials tested. Laser machining was also characterized by a proportional decrease in joint strength with increasing overlap size (Figure 16b).

**Figure 17:**
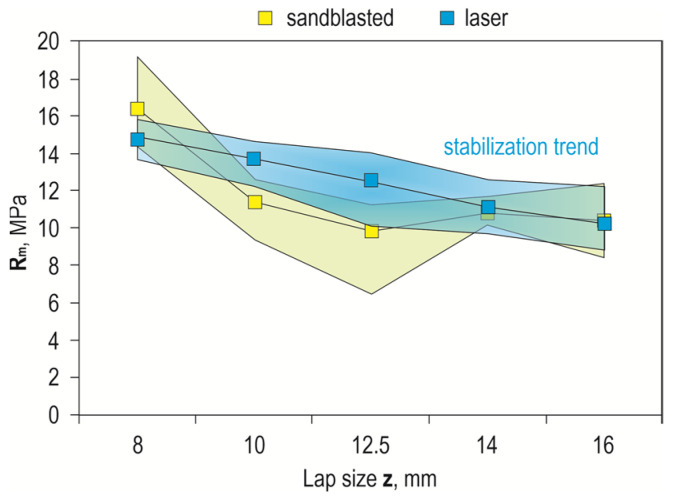
Comparison of the effects of sandblasting and laser treatment on the deviation from the mean value of lap joint strength (EN AW-2024).

Comparing the strength of the joints in terms of the tested material, the largest deviations were observed for the EN AW-2024 alloy, but laser treatment remains more favorable than sandblasting (Figure 17) due to the greater repeatability of the results obtained, which, in turn, is due to the invariability of the energy effect over time and the possibility of setting the machining parameters precisely. On the other hand, a problem that occurs during sandblasting is the possibility of uneven machining intensity. In addition, post-treatment with a laser makes it possible to impart directionality and repeatability to the arrangement of the resulting pits, which have been intentionally directed perpendicular to the direction of the breaking force. Compared to the random structure after sandblasting, this is definitely more advantageous.

**Figure 18:**
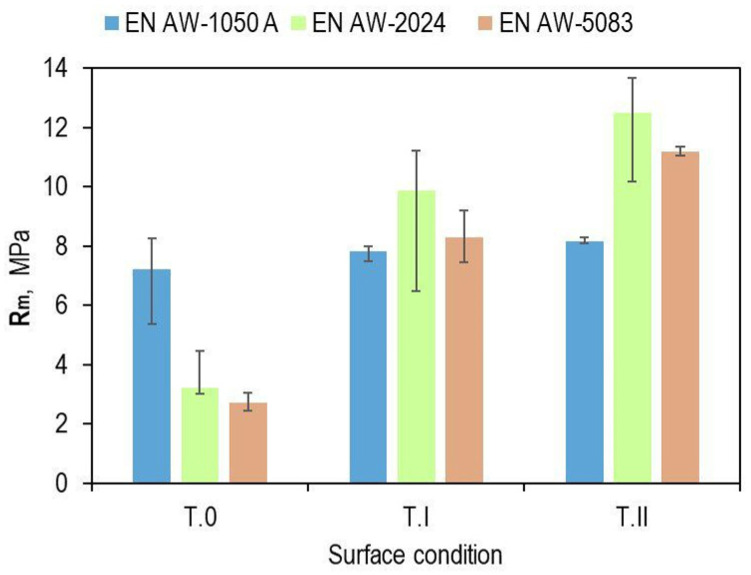
Effect of the surface condition of a 12.5 mm overlap on joint strength for different materials.

The smallest effect of the surface condition before bonding was observed for EN AW-1050A aluminum, while the largest was observed for the EN AW-2024 alloy, and a moderate effect was observed for the EN AW-5083 alloy, but with a trend in favor of laser treatment (Figure 18).

### 3.3. Surface Characterization

**Figure 19:**
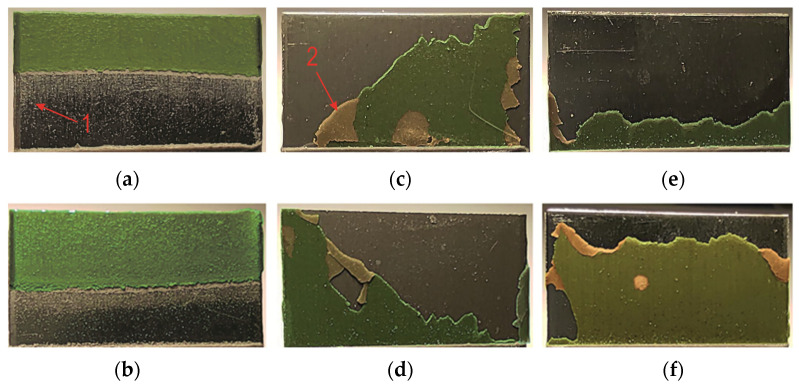
Adhesive structure on the surface of the overlap after cracking the joint (surface degreased with C_6_H_14_ compound—variant T.0): (**a**,**b**) EN AW-1050A; (**c**,**d**) EN AW-2024; (**e**,**f**) EN AW-5083.

After the overlap strength tests, macroscopic observations were made of the adhesive joint separation surfaces with Araldite 2014-2. The comparative analysis was performed for a standard overlap size of *z* = 12.5 mm. The adhesion forces on the surface of the EN AW-1050A alloy were high enough that the adhesive layer remained on both of the overlap surfaces in a similar surface proportion (Figure 19a,b). The exposed portion of the overlap surface shows microareas of remaining adhesive (detail “1” in Figure 19a) (cohesive bonding). In the case of the other two sheet materials (EN AW-2024 and EN AW-5083), an irregular border of adhesive layer damage was formed (Figure 19c–f). The brighter areas of the adhesive layer (detail “2” in Figure 19c) refer to the case of separation from both surfaces of the overlap in the joint. The cohesion forces of the adhesive material were high enough that these fragments remained unseparated from the remaining adhesively bonded adhesive layer.

**Figure 20:**
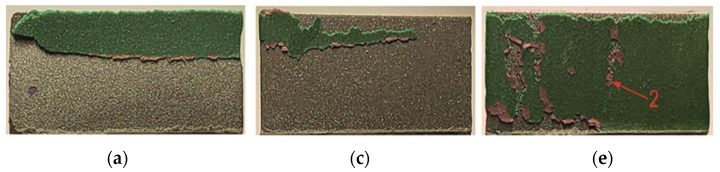

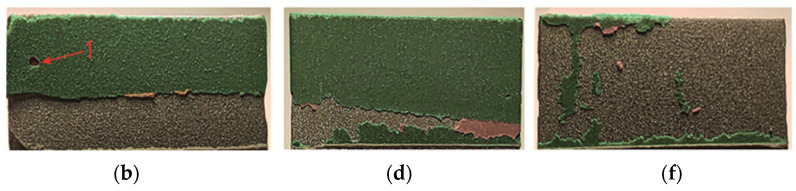
Adhesive structure on the surface of the overlap after the joint has been cracked (surface treated with sandblasting—variant T.I): (**a**,**b**) EN AW-1050A; (**c**,**d**) EN AW-2024; (**e**,**f**) EN AW-5083.

A view of the surface of the sandblasted overlap after the shear test of the joint is shown in Figure 20. In the case of the joint with EN AW-1050A, the disconnection of the sheets occurred due to the loss of adhesion of the adhesive to the surface of the sheets (Figure 20a,b). In one case, a loss of adhesive (detail “1” in Figure 20b) occurred on both surfaces of the overlap. In the other two cases of the substrate material, local glue detachments (detail “2” in Figure 20e) occurred on both surfaces of the sheets (Figure 20c–f). The limit of loss of cohesion of the adhesive layer that remained on the overlap surfaces was the longest for the substrate from EN AW-5083 (Figure 20e,f). 

**Figure 21:**
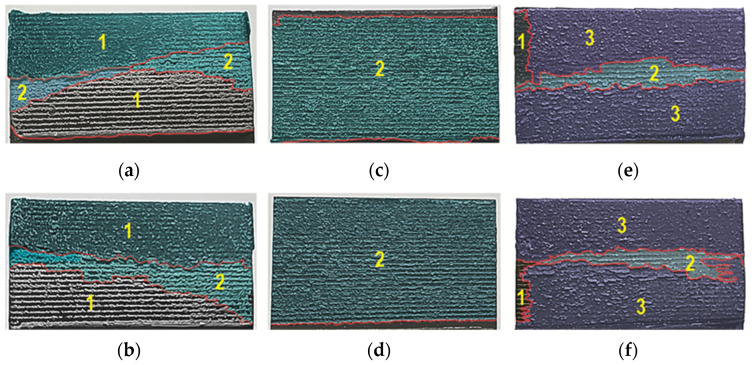
Adhesive structure on the surface of the overlap after joint fracture (laser-treated surface—variant T.II): (**a**,**b**) EN AW-1050A; (**c**,**d**) EN AW-2024; (**e**,**f**) EN AW-5083 (1—area of separation of the adhesive layer from the substrate/adhesive failure; 2—area of the surface with mixed adhesive-cohesive failure; 3—area of cohesive adhesive separation).

**Figure 22:**
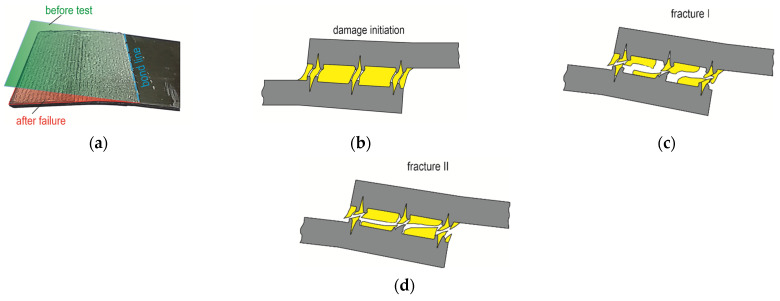
Plastic deformation of the adhesive substrate after separation of the lap joint (**a**); the onset of crack formation (**b**); I case of final cracking (**c**); II case of separation of the lap joint (**d**).

Laser treatment of the tested materials showed that EN AW-5083 sheets showed the greatest effect in terms of increased adhesive load-bearing capacity (Figure 17, Figure 21e,f, and Figure 22d). In the case of the EN AW-2024 alloy, there was a mixed failure mechanism over the entire overlap surface through loss of bond strength between the adhesive particles and the substrate and cohesion forces of the adhesive particles (Figure 21c–f and Figure 22c). An increased proportion of cohesive joint separation in the adhesive layer occurred for the EN AW-5083 alloy only (Figure 22d). It was observed that for the EN AW-1050A material, the onset of microcrack propagation occurred in the direction from the edges of the overlap to the center of the overlap (detail “1” and “2” in Figure 21a,b). Laser-treated surfaces, according to the specified texturing scheme, and not in all cases of the substrate material for the adhesive, showed a significant increase in load-bearing capacity (Figure 12a and Figure 18) but had the effect of increasing the total joint failure energy (Figure 13).

**Figure 23:**
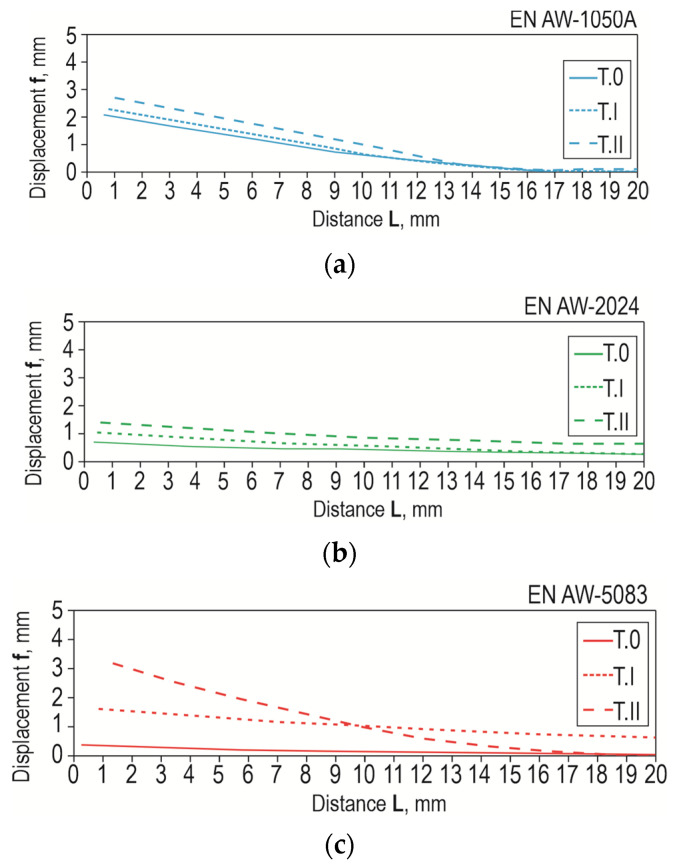
Effect of material grade and overlap surface treatment on the displacement of samples from the initial position for the material: (**a**) EN AW-1050A; (**b**) EN AW-2024; (**c**) EN AW-5083.

Laser texturing according to a specific pattern resulted in a specific pattern of microcracks, changing the bonding forces between the adhesive and the substrate (Figure 21 and Figure 22b–d). The deviation of the bonded surfaces from the initial position, using the 12.5 mm overlap as an example, is shown in Figure 23. The largest difference in the deviation of the overlap surface was observed for the EN AW-5083 alloy (Figure 23c). Out of the tested materials, the largest difference between the non-textured and laser-treated overlap occurred for EN AW-5083, while the smallest deviation from the initial position of the overlap occurred for joints with EN AW-2024 (Figure 23b). Relatively large bending of the overlap surface occurred for all three surface states (T.0, T.I, and T.II) for the joint with EN AW-1050A (Figure 23a). Of the three used, this alloy has the lowest mechanical properties (Table 2).

Although the EN AW-1050A material had a lower stiffness (lower *E* value), the bending of the overlap after laser treatment was less than for the EN AW-2024 material and greater than the joint from the EN AW-5083 material (Figure 23). For the three tested materials, the best result in terms of reproducible changes in the resulting overlaps after laser surface modification was obtained for the EN AW-5083 alloy (Figure 11). Repeated depths and overflows of the material resulted in a change in the occurrence of the adhesion layer material failure mechanism (Figure 22). The occurrence of regular material overbending contributed to the inhibition of the adhesive failure mechanism of the bonded joint. The increased bending of the sheets results from the EN AW-5083 material is due to the resulting the largest transverse material overblows. This can be confirmed by the bending lines as a function of distance from the beginning of the overlap (Figure 23).

## 4. Conclusions

This paper presents the results of an experimental study on the possibility of increasing the load-bearing capacity of a joint with an epoxy adhesive by using classical abrasive and laser treatment. Post-treatment surface bonding tests were performed without the use of harmful chemical cleaners and the overlap that modifies the surface. Analysis of the effectiveness of the use of laser surface treatment and the other two cases of surface modification was performed for three different aluminum alloys: EN AW-1050A, EN AW-2024, and EN AW-5083. The lowest roughness parameter (*Ra* and *Rz*) of the non-textured surface had the substrate of the overlap with EN AW-5083. This was reflected in the fact that the adhesive bond for this material showed the lowest load-bearing capacity. The chief conclusions of this study can be drawn as follows:

(1) The most reproducible shape of the material outflow at the edge of the created gap after laser treatment was obtained for the substrate from EN AW-5083. In this material, the gap was also the widest. The largest variation in the irregularity of the melt overflow occurred for the substrate with EN AW-1050A. The molten material moved back into the microcavity formed. The formed changes had the lowest repeatability.
(2) Despite the preservation of the initial surface condition (without additional chemical cleaning) between the laser-textured grooves, the load-bearing capacity of the joint (the average maximum force that the joint can carry) was 10% for EN AW-1050A, 285% for EN AW-2024, and 296% for EN AW-5083.
(3) Joints from EN AW-1050A showed the highest values of total joint failure energy. The mentioned material has the lowest yield strength. Adhesion bonds formed between the adhesive phase and the substrate were so strong that gradual bending of the overlap occurred during loading. As a result of the bending of the overlap in the adhesive layer, a normal force was generated in addition to the tangential force. The work of these forces during the load test of laser-treated joints was smallest for EN AW-2024.
(4) Joints with EN AW-1050A, the surfaces of which were prepared via sandblasting, showed a strength (*R_m_*) decreasing with the increasing overlap size. For the other two alloys, EN AW-2024 and EN AW-5083 had the lowest strength with a 12.5 mm overlap. In all cases, joints with an 8 mm overlap had the highest strength.

## Figures and Tables

**Table 1:** Chemical composition of aluminum alloy sheets. Maximum percentage by weight, % (according to [44]).

Material Designation	Element (Max Value)									
	Mg	Mn	Fe	Si	Cu	Zn	Cr	Ti	Zr+Ti	Others
EN AW-1050A	0.05	0.05	0.4	0.25	0.05	0.07	-	0.05	-	0.03
EN AW-2024	1.8	0.9	0.5	0.5	4.9	0.25	0.1	0.15	0.2	0.15
EN AW-5083	4.9	1.0	0.4	0.4	0.1	0.25	0.25	0.15	-	0.15

**Table 2:** Mechanical properties of aluminum alloy sheets [45].

Material Designation	Heat-Treated Condition	Brinell Hardness, HB	Young’s Modulus *E*, GPa	Poisson’s Ratio *ν*	Yield Strength *R_p0.2_* (min.), MPa	Tensile Strength *R_m_* (min.), MPa	Elongation after Fracture *A_50_*, %
EN AW-1050A	H24	33	69	0.33	75	105	4
EN AW-2024	T351	120	74	0.33	290	435	20
EN AW-5083	H111	75	71	0.33	125	275	12

**Table 3:** Mechanical properties of fully cured Araldite 2014-2 adhesive (mixed 2:1).

Adhesive	Young Modulus *E*, GPa	Shear Modulus *G*, GPa	Tensile Yield Strength *R_m_*, MPa	Shear Yield Strength, MPa	Poisson Ratio, ν	Elongation at Break, %
Araldite 2014-2	3.1	1.2	30	22	0.33	0.9

**Table 4:** Chemical composition of micropowder of electrocorundum F280 (%).

Al_2_O_3_	SiO_2_	Fe_2_O_3_	Na_2_O
99.20	0.08	0.06	0.35

**Table 5:** Summary of tested combinations of single-lap joints (x—made variant).

Material Grade	Lap *z*, mm	Surface Treatment		
		Without Texturing T.0	Texturing	
			Electrocorundum T.I	Laser T.II
EN AW-1050A	8	-	x	x
	10	-	x	x
	12.5	x	x	x
	14	-	x	x
	16	-	x	x
EN AW-2024	8	-	x	x
	10	-	x	x
	12.5	x	x	x
	14	-	x	x
	16	-	x	x
EN AW-5083	8	-	x	x
	10	-	x	x
	12.5	x	x	x
	14	-	x	x
	16	-	x	x

**Table 6:** The statistical parameters obtained from the experimental data (without texture).

Parameter	Material	Values of Parameters, μm			
		Standard Value	Mean Deviation	Standard Deviation	Standard Deviation of the Mean Value
*Ra*	EN AW-1050A	0.186	0.171	0.005	0.002
	EN AW-2024	0.181	0.176	0.004	0.001
	EN AW-5083	0.136	0.131	0.003	0.002
*Rz*	EN AW-1050A	1.681	1.495	0.038	0.017
	EN AW-2024	2.341	2.123	0.012	0.008
	EN AW-5083	1.28	1.16	0.006	0.003

## Data Availability

Data are contained within the article.
